# Calcium channel β3 subunit regulates ATP-dependent migration of dendritic cells

**DOI:** 10.1126/sciadv.adh1653

**Published:** 2023-09-20

**Authors:** Marcel S. Woo, Friederike Ufer, Jana K. Sonner, Anouar Belkacemi, Joseph Tintelnot, Pablo J. Sáez, Paula F. Krieg, Christina Mayer, Lars Binkle-Ladisch, Jan Broder Engler, Simone Bauer, Nina Kursawe, Vanessa Vieira, Stefanie Mannebach, Marc Freichel, Veit Flockerzi, Pablo Vargas, Manuel A. Friese

**Affiliations:** ^1^ Institute of Neuroimmunology and Multiple Sclerosis, University Medical Center Hamburg-Eppendorf, 20251 Hamburg, Germany.; ^2^ Institute of Pharmacology, Heidelberg University, 69120 Heidelberg, Germany.; ^3^ Experimental and Clinical Pharmacology and Toxicology, Saarland University, 66421 Homburg, Germany.; ^4^ Institut Curie and Institut Pierre-Gilles de Gennes, PSL Research University, CNRS, UMR 144, F-75005, Paris, France.; ^5^ Cell Communication and Migration Laboratory, Institute of Biochemistry and Molecular Cell Biology, University Medical Center Hamburg-Eppendorf, 20246 Hamburg, Germany.; ^6^ DZHK (German Centre for Cardiovascular Research), partner site Heidelberg/Mannheim, 69120 Heidelberg, Germany.; ^7^ Université Paris Cité, INSERM UMR-S1151, CNRS UMR-S8253, Institut Necker Enfants Malades, Paris, France.

## Abstract

Migratory dendritic cells (migDCs) continuously patrol tissues and are activated by injury and inflammation. Extracellular adenosine triphosphate (ATP) is released by damaged cells or actively secreted during inflammation and increases migDC motility. However, the underlying molecular mechanisms by which ATP accelerates migDC migration is not understood. Here, we show that migDCs can be distinguished from other DC subsets and immune cells by their expression of the voltage-gated calcium channel subunit β3 (Cavβ3; CACNB3), which exclusively facilitates ATP-dependent migration in vitro and during tissue damage in vivo. By contrast, CACNB3 does not regulate lipopolysaccharide-dependent migration. Mechanistically, CACNB3 regulates ATP-dependent inositol 1,4,5-trisphophate receptor–controlled calcium release from the endoplasmic reticulum. This, in turn, is required for ATP-mediated suppression of adhesion molecules, their detachment, and initiation of migDC migration. Thus, *Cacnb3*-deficient migDCs have an impaired migration after ATP exposure. In summary, we identified CACNB3 as a master regulator of ATP-dependent migDC migration that controls tissue-specific immunological responses during injury and inflammation.

## Teaser

The calcium channel β3 subunit fine-tunes calcium release from the endoplasmic reticulum and regulates ATP-induced DC migration.

## INTRODUCTION

Dendritic cells (DCs) are antigen-presenting cells that orchestrate the immune response by constantly patrolling the entire body. The subpopulation of migratory DCs (migDCs) is localized at host-environment interfaces. migDCs are derived from the bone marrow and populate different organs of the body as precursors from the blood stream where they undergo permanent homeostatic maturation (*1*). They have the important ability to migrate from tissues to lymphoid organs through lymphatic vessels. In particular, at body surfaces, they take up and subsequently carry processed antigens to present them to effector immune cells in the secondary lymphoid organs (*2*, *3*). Their antigen sampling and presentation as well as activation state and migratory speed are modified by the presence of pathogen-derived cues or host-derived danger signals (*4*, *5*). During infection and inflammation, migDCs rapidly mature and accelerate migration from their respective tissues to eventually enter draining lymph nodes (dLNs) through the lymphatic vessels to interact with T cells for antigen presentation. However, little is how tissue damage shapes migration and its context-dependent induction or inhibition of directed adaptive immune responses (*6*).

In this context, we previously identified the neuronal plasticity molecule activity-regulated cytoskeleton-associated protein (Arc/Arg3.1) as a specific regulator of DC migration in response to the pathogen-associated molecular pattern (PAMP) lipopolysaccharide (LPS) (*7*). However, tissue damage and inflammation equally shape DC migration by release of a variety of danger signals from damaged cells or through secretion by activated parenchymal or infiltrating immune cells (*8*). A key damage-associated molecular pattern (DAMP) is extracellular adenosine triphosphate (ATP) that affects migDC migration and coordinates the innate immune response in the vicinity of tissue damage by binding to ionotropic P2X receptors (P2RXs) and metabotropic P2Y receptors (P2RYs) (*9*, *10*). Activation of P2RXs induces an increase in cytosolic calcium and sodium concentrations. Especially well studied are P2RX4 and P2RX7 that, upon activation, induce proinflammatory phenotypes in T cells, macrophages, and monocytes (*11*, *12*). ATP binding to P2RYs induces a G protein–coupled signaling cascade that results in inositol 1,4,5-trisphophate (IP_3_) receptor (IP_3_R)–dependent calcium release from the endoplasmic reticulum (ER) into the cytosol, which can act both pro- and anti-inflammatory (*13*, *14*). For example, activation of P2RY6 inhibits effector T cell activation in a mouse model of pulmonary inflammation (*15*), whereas P2RY14 promotes signal transducer and activator of transcription 1–dependent secretion of inflammatory cytokines in macrophages (*16*, *17*). Because of the immunomodulatory impact of ATP, its concentration is tightly controlled and actively being kept in a nanomolar range by the exonucleotidases CD39 and CD73 that cleave ATP and generate adenosine (ADO) (*18*). The latter binds to ADO receptors that dampen inflammatory phenotypes and, for example, foster the differentiation of regulatory T cells and homeostatic microglia (*19*–*21*). Therefore, while homeostatic levels of ATP and ADO are well balanced, abundant release of ATP will influence the outcome of inflammation and tolerance.

Similarly, migDCs express high levels of a variety of P2RXs, P2RYs, and ADO receptors as well as CD39 and CD73 (*2*, *22*, *23*). ATP activates P2RX7 that results in pannexin-1–dependent release of ATP inducing an autocrine loop that augments chemotaxis of migDCs. Engagement of P2RX7 by increased ATP levels induces cytosolic calcium levels that result in a rearrangement of the actin cytoskeleton and accelerated migration (*22*, *24*). However, inhibition the P2RX7 in migDCs does not completely inhibit fast migration (*22*). Thus, besides the pannexin-1–P2RX7 axis, additional ATP-induced signaling cascades have to be present to regulate migDC migration. In particular, the role and molecular regulators of the ATP-induced calcium currents are unknown.

Here, we set out to define exclusive determinants of migDC function and identified the voltage-gated calcium channel auxiliary subunit β3 (CACNB3) as master regulator of ATP-dependent migration. By contrast, other regulatory and pore-forming subunits of voltage-gated calcium channels were not expressed in migDCs. By measuring the impact of CACNB3 on DC migration in vitro and in vivo during tissue damage and inflammation, we found that it selectively regulates ATP-dependent migration in contrast to pathogen-induced migration. Mechanistically, we detected that CACNB3 regulates ATP-induced calcium release from the ER and thereby enables down-regulation of surface adhesion molecules with subsequent detachment and initiation of migration. Together, we identified a mechanism that specifically modulates ATP-dependent migration in migDCs, which could be used to directly modulate this process in inflammatory diseases.

## RESULTS

### CACNB3 expression defines migDCs

**Fig. 1.**
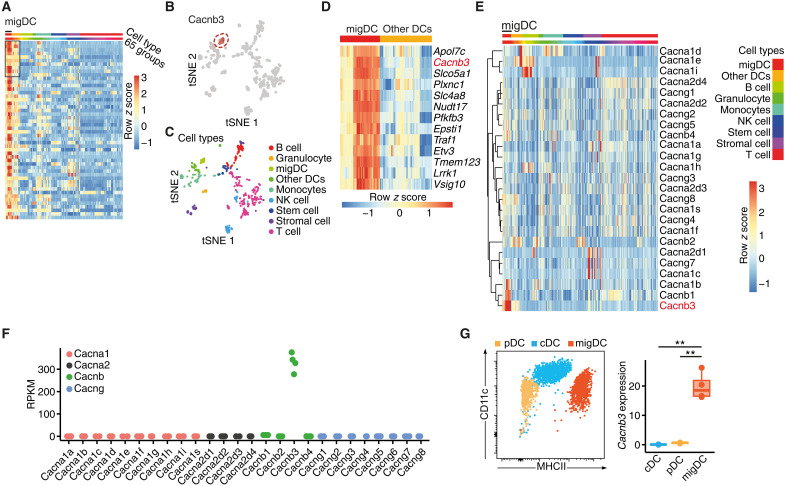
CACNB3 is exclusively expressed in migDCs. (**A**) Heatmap showing differential expression analysis of migDCs against 64 other immune cell groups retrieved from the ImmGen database (GSE15907). Rows depict genes sorted by the highest expression in migDCs in comparison to all other cell types, columns are grouped by cell types, and color shows row *z* score. (**B**) *t*-distributed stochastic neighbor embedding (tSNE) plot depicting each of the 653 samples included in the ImmGen database (GSE15907), labeled are all Cacnb3-high expressing samples. Color shows relative expression. NK cell, natural killer cell. (**C**) tSNE plot depicts each sample, and colors define cell types. (**D**) Heatmap of the top 13 genes that are differentially expressed in migDCs in comparison to each other cell type sorted by average log fold change. Color shows row *z* score. (**E**) Heatmap showing all voltage-gated calcium channel subunits. Hierarchical clustering was performed for rows, and color shows row *z* score. (**F**) Reads per kilobase of transcript per million mapped reads (RPKM) of voltage-gated calcium channel subunits from sorted migDCs that were used for RNA sequencing (*n* = 4). (**G**) *Cacnb3* RNA expression of sorted plasmacytoid DCs (pDCs), classical DCs (cDCs), and migDCs from sdLNs (*n* = 3). Unpaired *t* test was used for statistical comparison, **P < 0.01.

Following up on our previous work (*7*), we set out to further characterize unique functions of migDCs. To identify exclusively expressed genes of migDCs that could give deeper insights into their function, we first mined the publicly available ImmGen database (*25*–*27*). We compared the transcriptomes of LN-resident migDCs (fig. S1A) against all other 64 immune cell subsets available (Fig. 1, A to C), which resulted in 13 genes that were exclusively expressed in migDCs in comparison to other DCs (Fig. 1D). The most distinct gene transcripts were apolipoprotein 7c (*Apol7c*) and the voltage-gated CACNB3 (*Cacnb3*). Confirmatory, migDCs highly expressed the *Arc/Arg3.1* gene, which we previously identified as specific marker for migDCs (fig. S1B) (*7*), and they were enriched for the Gene Ontology (GO) term “antigen processing and antigen presentation” (fig. S1C). We further focused on *Cacnb3* since this gene is highly conserved and shared among species, whereas no human orthologue exists for *Apol7c*. A detailed analysis of *Cacnb3* expression in different DC subsets revealed the highest expression in MHCII^high^Langerin^−^CD103^+^CD11b^+^, MHCII^high^Langerin^−^CD103^+^CD11b^low^, MHCII^high^Langerin^+^CD103^−^CD11b^+^, and MHCII^high^Langerin^+^CD103^+^CD11b^low^ migDCs in skin dLNs (sdLNs) (fig. S1D). In contrast, skin-resident Langerhans cells showed a weak *Cacnb3* expression, suggesting a specific role for *Cacnb3* in migDC subsets originating from the bone marrow. Since CACNB3 is a subunit of voltage-gated calcium channels, we next analyzed the expression of the other subunits in the ImmGen database (Fig. 1E) and by RNA sequencing of sorted migDCs (Fig. 1F). Notably, migDCs exclusively expressed *Cacnb3* but none of the other regulatory and pore-forming subunits, which are required for membranous voltage-gated calcium channels. This was in contrast to neurons that highly expressed *Cacnb3* together with other subunits that are functionally related (fig. S1E). In addition, probing sorted migDCs, plasmacytoid DCs, and classical DCs from sdLNs for their *Cacnb3* transcript levels by quantitative polymerase chain reaction (PCR) confirmed the exclusive *Cacnb3* expression in migDCs (Fig. 1G and fig. S1, F and G). Together, we concluded that *Cacnb3* expression defines migDCs and asked whether CACNB3 expression relates to a unique function in this important regulatory immune cell subset.

### CACNB3 specifically regulates ATP-dependent migration

**Fig. 2.**
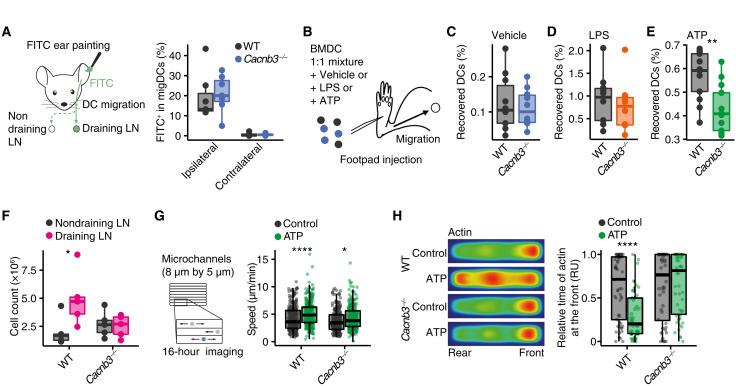
*Cacnb3*-deficient migDCs show an impaired ATP-dependent migration. (**A**) FITC ear painting in WT and *Cacnb3^−/−^* (*n* = 7) mice and quantification of FITC^+^ migDCs in ipsilateral and contralateral cervical LNs. (**B** to **E**) Graphical summary (B) and quantification of recovered BMDCs from draining ipsilateral popliteal LNs of WT and *Cacnb3^−/−^* BMDCs that were mixed in a 1:1 ratio and pulsed with vehicle (C) (WT, *n* = 9; *Cacnb3^−/−^ n* = 8), LPS (100 ng/ml) (D) (WT, *n* = 9; *Cacnb3^−/−^*, *n* = 7), or 500 μM ATP (E) (WT, *n* = 10; *Cacnb3^−/−^*, *n* = 10) and injected into the footpad of WT mice. (**F**) Quantification of migDCs in WT- and *Cacnb3^−/−^*-draining and nondraining cervical LNs after ear tape stripping (*n* = 5). (**G**) Quantification of mean speed (in micrometers per minute) of WT and *Cacnb3^−/−^* BMDCs that were imaged in microchannels after pulsing with vehicle or 500 μM ATP. (**H**) Quantification of relative time of actin at the front of WT and *Cacnb3^−/−^* BMDCs [in relative units (RU)] that were imaged in microchannels after pulsing with vehicle or 500 μM ATP. Unpaired *t* test was used for statistical comparison, *P < 0.05, **P < 0.01, and ****P < 0.0001.

Because CACNB3 is exclusively expressed in migDCs, we next investigated whether it regulates DC migration. We first compared the migration of wild-type (WT) and *Cacnb3*-deficient (*Cacnb3^−/−^*) migDCs in vivo using skin painting with fluorescein isothiocyanate (FITC). Painting the ear skin of mice with FITC allows to assess the uptake of FITC by migDCs and their inflammation-driven mobilization and migration to the dLNs. Notably, no differences were measured for both genotypes when comparing the number of FITC-positive migDCs that had migrated to the ipsilateral dLNs that served as control (Fig. 2A). Next, we aimed to evaluate the capacity of DAMP- or PAMP-activated migDCs to migrate to dLNs in vivo. For this, we injected into the footpads of WT mice an even mixture of WT and *Cacnb3^−/−^* bone marrow–derived DCs (BMDCs) after ex vivo exposure to vehicle, 500 μM ATP, or LPS (100 ng/ml) for 30 min and measured the recovered BMDCs in the draining popliteal LNs (Fig. 2B) by flow cytometry. Again, we found no differences in vehicle-stimulated (Fig. 2C) or LPS-stimulated (Fig. 2D) BMDCs. By contrast, we recovered significantly less *Cacnb3^−/−^* BMDCs from dLNs after ATP stimulation (Fig. 2E). Likewise, after LPS stimulation, we detected no differences in activation marker induction on WT and *Cacnb3^−/−^* BMDCs (fig. S2, A to E).

To further investigate the ATP-dependent migration, we used ear tape stripping as a model for subtle tissue injury that results in local ATP release (*28*). Notably, we detected an increased migration of WT but not *Cacnb3^−/−^* migDCs into ipsilateral draining cervical dLNs after ear tape stripping (Fig. 2F and fig. S2F). In particular, the relative frequency of different migDC subsets was not affected after ear tape stripping in the absence of CACNB3 (fig. S2G). Next, we analyzed the migration of WT and *Cacnb3^−/−^* BMDCs in microchannels after 30 min of 500 μM ATP treatment. Consistently, WT BMDCs showed a stronger increase in average speed after ATP stimulation than *Cacnb3^−/−^* BMDCs (Fig. 2G and fig. S2H). Again, no differences in acceleration were observed between WT and *Cacnb3^−/−^* BMDCs after LPS stimulation (fig. S2, I and J). Actin redistribution that is required for migration was also disturbed after ATP stimulation, as we observed a diminished relocation of actin from the front to the rear end in *Cacnb3^−/−^* BMDCs in comparison to WT BMDCs (Fig. 2H). Notably, when we analyzed WT and *Cacnb3^−/−^* mice in steady state, we did not detect any a priori differences between genotypes in immune cell numbers or subset composition in the blood, spleen, aortic LNs, or sdLNs (fig. S3, A and B). A more detailed analysis of migDC subsets and Langerhans cell frequencies in sdLN of *Cacnb3^−/−^* and WT mice also showed no differences between genotypes (fig. S3C). Likewise, the expression of the ADO exonucleotidases CD39 and CD73 in various immune cell subsets was unaltered in sdLNs and spleen in the absence of CACNB3 (fig. S3, D to G). Thus, CACNB3 specifically regulates migration of migDCs in response to the danger signal ATP, and *Cacnb3^−/−^* mice do not show any a priori immune cell deficiencies.

### ATP-induced calcium release from the ER is modulated by CACNB3

**Fig. 3.**
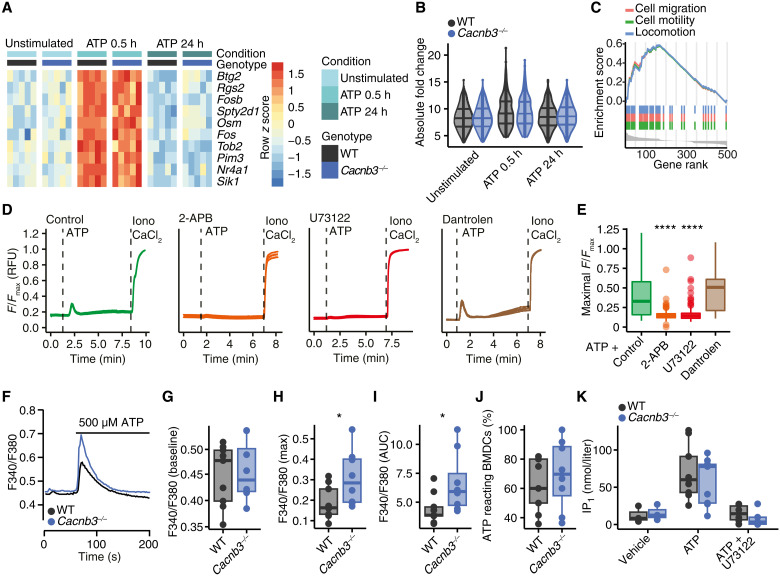
IP_3_R-mediated calcium release is inhibited by CACNB3. (**A**) Heatmap of identified genes by RNA sequencing in WT and *Cacnb3^−/−^* BMDCs 30 min or 24 hours after a 30-min pulse of 500 μM ATP (*n* = 5). Color shows row *z* score. (**B**) Absolute log_2_ fold change of top 50 regulated genes between WT and *Cacnb3^−/−^* BMDCs across all conditions. (**C**) GO term enrichment of cell migration [normalized enrichment score (NES) = 1.52, *P*adj = 0.003], cell motility (NES = 1.52, *P*adj = 0.003), and locomotion (NES = 1.52, *P*adj = 0.002) in WT BMDCs 30 min after ATP pulse. (**D** and **E**) Representative means of calcium traces with SEM (D) and quantification (E) of WT BMDCs that were stimulated with 500 μM ATP and pretreated for 10 min with vehicle (*n* = 214), 2-APB (*n* = 113), U73122 (*n* = 167), or dantrolene (*n* = 54). (**F**) Mean Fura-2 (F340/F380) ratiometric traces from WT and *Cacnb3^−/–^*BMDCs before and after the addition of 500 μM ATP. (**G** to **J**) Quantification of resting calcium levels (G), calcium peak (H), and AUC after the addition of 500 μM ATP (I) and percentage of BMDCs that reacted to 500 μM ATP application with increase in cytosolic calcium levels (J) in WT (*n* = 9) and *Cacnb3^−/−^* (*n* = 8) BMDCs. (**K**) IP_1_ concentration (in nanomoles per liter) measured in WT and *Cacnb3^−/−^* BMDCs before and after the addition of 500 μM ATP in the absence or presence of 10 μM U73122 as indicated (*n* = 3 to 9 wells). Data are shown as single values and median ± interquartile range. All calcium imaging experiments were performed in calcium-free media. If not stated otherwise, unpaired two-tailed Wilcoxon-Mann-Whitney test was used for statistical comparison, *P < 0.05 and ****P < 0.0001.

After having established that CACNB3 is required for ATP-induced migration of migDCs, we interrogated the underlying mechanism. To identify potential transcriptional changes that are imposed by CACNB3, we sequenced the RNA of WT and *Cacnb3^−/−^* BMDCs in an unstimulated state as well as 30 min and 24 hours after ATP pulse stimulation. Notably, we detected no differentially regulated genes between genotypes at any time point (Fig. 3, A and B, and fig. S4, A to C) including P2RX and P2RY (fig. S4D). However, reassuringly after ATP stimulation, both genotypes showed an increase in the GO terms “cell migration,” “cell motility,” and “locomotion” on transcriptional level (Fig. 3C and fig. S4, E and F).

Because CACNB3 is not involved in transcriptional adaptation and previous studies demonstrated that ATP-induced acceleration of migration depends on calcium release from the ER (*24*), we next monitored intracellular calcium changes by live-cell imaging. We conducted these experiments in BMDCs under calcium-free conditions and pharmacologically blocked the two main receptors that regulate ER calcium release, IP_3_R, and ryanodine receptors (*29*). ATP stimulation increased cytosolic calcium levels in BMDCs that we could inhibit by preincubation with the broad IP_3_R and store-operated calcium entry (SOCE) blocker 2-aminoethoxydiphenyl borate (2-APB) and the phospholipase C (PLC) antagonist U73122 but not with the ryanodine receptor inhibitor dantrolene (Fig. 3D). In addition, preincubation with the inhibitor of the sarcoplasmic reticulum calcium adenosine triphosphatase thapsigargin completely abolished the ATP-induced calcium response (fig. S5, A and B), supporting our finding that ATP induces IP_3_R-dependent calcium release from the ER in BMDCs (Fig. 3E).

We tested whether CACNB3 directly modulates IP_3_R activation, as we have recorded for fibroblasts (*30*). Notably, the IP_3_R isotypes and PLC isotypes (fig. S5, C and D) were not differently expressed in WT and *Cacnb3^−/−^* BMDCs. We measured the cytosolic calcium levels (example traces are shown in Fig. 3F) by loading BMDCs with either the ratiometric fluorophore Fura-2 or the nonratiometric fluorophore Fluo-4. Baseline calcium levels during steady state (Fig. 3G) and cytosolic levels after external calcium application or after isolated activation of SOCE by subsequent calcium chelation with EDTA and addition of external calcium were not different between WT and *Cacnb3^−/−^* BMDCs (fig. S5E). By contrast, after treatment with 500 μM ATP, we recorded an increased calcium peak (Fig. 3H and fig. S5, F and G) and cumulative area under the curve (AUC; Fig. 3I) in *Cacnb3^−/−^* BMDCs in comparison to WT BMDCs, while the percentage of ATP-reactive BMDCs was similar (Fig. 3J), with a return to comparable baseline calcium levels 30 min after ATP stimulation (fig. S5F). In contrast, the ATP-induced levels of inositol 1-phosphate (IP_1_), which is the stable metabolite of IP_3_ and serves as an indicator for the enzymatic activity of PLCs, did not differ between WT and *Cacnb3^−/−^* BMDCs (Fig. 3K), underlining that CACNB3 does not modulate upstream signaling of the IP_3_R. Moreover, we did not detect differences in induction of pannexin-1–induced membranous macropores, which we concluded from measuring ATP-induced 4′,6-diamidino-2-phenylindole (DAPI) uptake (fig. S5, H and I) (*22*). Together, CACNB3 limits IP_3_R-mediated calcium release after ATP stimulation in migDCs that does not require transcriptional changes.

### Calcium release from the ER modulates surface expression of adhesion molecules

**Fig. 4.**
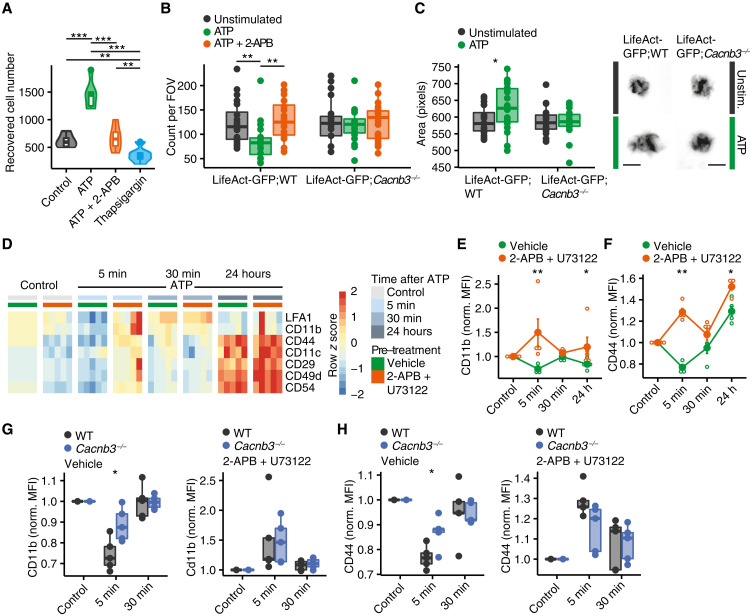
CACNB3-regulated calcium release from the ER regulates DC detachment. (**A**) Recovered cell number of BMDCs (*n* = 6) in a transwell cell migration assay with CCL-19/CCL-21 as chemoattractant 16 hours after indicated stimulation after a 30-min pulse with 500 μM ATP, 500 μM ATP, 50 μM 2-APB, and 1 μM thapsigargin. (**B**) Count of WT and *Cacnb3^−/−^* BMDCs per field of view (FOV) in fibronectin-coated wells 6 hours after a 30-min pulse with 500 μM ATP or 500 μM ATP with 50 μM 2-APB (*n* = 20). (**C**) Area (in pixels) of BMDCs from *LifeAct-GFP*;WT and *LifeAct-GFP*;*Cacnb3^−/−^* mice 6 hours after a 30-min pulse with 500 μM ATP (*n* = 20). (**D**) Heatmap of selected surface molecule expression of indicated proteins measured by flow cytometry at baseline, 5-min ATP stimulation, 30-min ATP stimulation, and 24 hours after a 30-min ATP pulse. Color shows row score, and columns are grouped by time point of stimulation and vehicle or 2-APB and U73122 before treatment. Data were normalized to untreated controls. (**E** and **F**) Normalized (norm.) mean fluorescence intensity (MFI) of CD11b and CD44 (F) at baseline, 5-min ATP stimulation, 30-min ATP stimulation, and 24 hours after a 30-min ATP pulse with and without 2-APB and U73122 before treatment (*n* = 5). Data were normalized to controls of each genotype. (**G** and **H**) Normalized MFI of CD11b (H) and CD44 (I) of WT and *Cacnb3^−/−^* BMDCs at baseline, 5 min, and 30 min after ATP stimulation with vehicle or 2-APB and U73122 before treatment. Data were normalized to controls of each genotype. Unpaired *t* test was used for statistical comparison, *P < 0.05, **P < 0.01, and ***P < 0.001.

Because we detected CACNB3-dependent ATP-elicited calcium signals to be short-lived similar to what we have observed in fibroblasts (*30*), we next hypothesized that CACNB3 regulates detachment and initiation of migration. To test our hypothesis, we first made sure that in a transwell migration assay using C-C motif chemokine ligand 19 (CCL-19) and CCL-21 as chemoattractant, migration of WT BMDCs increased after 30 min of ATP pulse compared to vehicle-pulsed controls and was inhibited by 2-APB (Fig. 4A and fig. S6A). Notably, complete emptying of ER calcium stores by applying a 30-min pulse of thapsigargin inhibited transwell migration as well (Fig. 4A), supporting the notion that a balanced cytosolic calcium increase promotes migDC migration, whereas inhibited or excessive calcium release from the ER decreases migration. In addition, we crossed transgenic LifeAct mice that harbor green fluorescent protein (GFP)–coupled actin (*31*) to *Cacnb3^−/−^* mice (*LifeAct-GFP*;*Cacnb3^−/−^*) for our next experiments. We analyzed the likelihood of detachment of *LifeAct-GFP*;WT and *LifeAct-GFP*;*Cacnb3^−/−^* BMDCs by seeding them on fibronectin-coated surfaces and counting the attached BMDCs 6 hours after ATP stimulation in the absence or presence of 2-APB. We observed an increased detachment of *LifeAct-GFP*;WT BMDCs after ATP stimulation that was counteracted by 2-APB before treatment. By contrast, *LifeAct-GFP*;*Cacnb3^−/−^* BMDCs showed no increase in detachment after ATP stimulation (Fig. 4B). Notably, LPS stimulation induced a similar detachment of WT and *Cacnb3^−/−^* BMDCs (fig. S6B). When we quantified morphometric changes in BMDCs from *LifeAct-GFP*;WT mice, we found that ATP stimulation resulted in an elevated actin area of *LifeAct-GFP*;WT BMDCs with an increased directionality suggesting a detached migratory phenotype that was absent in *LifeAct-GFP*;*Cacnb3^−/−^* BMDCs (Fig. 4C and fig. S6, C to L).

After having recorded a functionally impaired ATP-induced detachment of *Cacnb3^−/−^* BMDCs, we measured the surface expression of adhesion molecules by flow cytometry. In WT BMDCs, after 5 min of ATP application, we recorded a strong decrease in adhesion molecule cell surface abundance that returned back to baseline after 30 min (Fig. 4D) and was inhibited by pretreatment of 2-APB and U73122 (Fig. 4, E and F, and fig. S7A). By contrast, *Cacnb3^−/−^* BMDCs showed an attenuated down-regulation of the adhesion molecules CD11b and CD44 in response to ATP, which again could be inhibited by prestimulation with 2-APB and U73122 (Fig. 4, G and H, and fig. S7, B and C). Notably, we detected no differences in baseline RNA and protein expression of adhesion molecules in the absence of CACNB3 (fig. S7, D and E). Thus, we concluded that CACNB3-regulated calcium transients after ATP stimulation decrease surface adhesion molecule expression of migDCs, which might allow them to detach and accelerate their migration.

## DISCUSSION

migDCs play a pivotal role in orchestrating the specificity and scale of adaptive immune responses. Their ability to detect endogenous danger signals and pathogens in tissues and subsequent migration to LNs via the lymphatics to instruct other immune cells requires a strictly regulated and stimulus-sensitive migratory machinery (*6*). Extracellular ATP is readily and abundantly increased during tissue injury and inflammation and induces calcium currents in DCs (*8*, *32*). This is followed by an autocrine loop via secretion of ATP by pannexin-1 channels that inform and further activate surrounding cells independently of cytosolic calcium levels (*22*). Here, we identified CACNB3 as key regulator of ATP-induced migDC migration and found that ATP elicited calcium release from the ER that decreases surface expression of adhesion molecules CD11b and CD44 and thereby initiates migration (*33*, *34*). This fine-tuned mechanism might be pivotal to balance immune responses in inflammatory tissues.

The exposure of migDCs to a variety of stimuli comes with the challenge to selectively regulate the subsequent immune cascade. We have previously identified Arc/Arg3.1 as key regulator of LPS-induced migration of migDCs (*7*). Here, we leveraged publicly available transcriptomics of sorted and defined immune cell subsets and found that migDCs exclusively express CACNB3. CACNB3 has been initially found and described as a regulatory β subunit of L-type calcium channels in neurons where it assembles with the α pore-forming subunits and other ancillary subunits (*35*–*37*). Furthermore, while *Cacnb3^−/−^* CD8^+^ T cells have impaired survival because of sustained calcium influx through voltage-gated calcium channel subunit alpha1 F (Ca_v_1.4) (*38*), migDCs do not express Ca_v_1.4 (encoded by *Cacna1f*), and, thus, CACNB3 ought to have another function in migDCs. We also found that migDCs do not express any other subunits suggesting a function that is distinct from pore-forming calcium channels as in other cell types for example neurons. *Cacnb3* deficiency resulted in an impaired ATP-induced migration, whereas baseline and LPS-mediated migration were not affected. This is particularly noteworthy as our previous findings showed impaired steady-state and LPS-induced migration in *Arc*-deficient BMDCs (*7*, *39*). The specific impairment of ATP-dependent migration is consistent with studies that showed different migratory patterns between PAMP and DAMP exposure (*40*).

Mechanistically, we detected that ATP elicited a cytosolic calcium response, which is in contrast to LPS stimulation. This is in line with other studies that have shown that an increase in intracellular calcium level is required for ATP-induced acceleration (*29*) and that chelation of intracellular calcium inhibits migration of migDCs (*22*). Furthermore, it has been shown that an increased frequency of IP_3_R-dependent calcium oscillations is associated with higher speed fluctuations in immature BMDCs (*24*). ATP activates the P2RX7, which results in calcium influx from the extracellular space (*22*). Here, we show that independent from extracellular calcium levels, ATP activates the PLC-IP_3_R signaling cascade, which results in calcium release from the ER. Thus, we propose that this is mediated by P2RY activation as has been shown for numerous other cell types (*41*–*43*). *Cacnb3* deficiency resulted in an unregulated release of calcium from the ER as has been shown for fibroblasts and pancreatic β cells where CACNB3 binds to IP_3_R in steady state and detaches after stimulation of G_q_-coupled G protein–coupled receptors (*30*, *44*). Another source of cytosolic calcium increase is activation of SOCE, which is the main source of calcium and activator of T cells (*45*, *46*). Notably, SOCE was unchanged in *Cacnb3*-deficient BMDCs that is concurrent with a previous study that showed a strong impairment of BMDC functionality after unselective calcium chelation but not after genetic deletion of *Stim1* and *Stim2* (*47*).

Furthermore, ATP can induce autocrine loops by secreting ATP through pannexin-1 after activation of P2RX7 in migDCs (*22*). This has also been shown for other P2RXs in different cells, for example, γδ T cells induce a similar ATP autocrine loop by P2RX4 stimulation (*48*). The pore-opening complexes can be measured by DAPI uptake after ATP stimulation. However, we did not find differences in DAPI uptake after ATP stimulation in *Cacnb3*-deficient BMDCs, and *Panx1* was not differently expressed between WT and *Cacnb3*-deficient BMDCs. Therefore, ATP-elicited PLC-IP_3_R–dependent calcium release is likely independent from the P2RX7-PANX1 axis. This is supported by the observation that *Panx1*-deficient BMDCs do not show differences in ATP-induced calcium responses and that P2RX7 inhibition does not fully block ATP-induced migration (*22*). We speculate that ATP-induced migDC migration is synergistically mediated by ionotropic ATP signaling through P2RX7 and metabotropic ATP signaling, which is fine-tuned by CACNB3.

Because of the temporal dynamics of fast calcium spikes after ATP stimulation, we hypothesized that calcium release regulates the detachment, which we validated by showing an IP_3_R-dependent decrease in adhesion molecule surface expression after ATP stimulation. Adhesion molecules can be regulated by changes in conformation or expression levels on the cell surface and recycling (*33*). These mechanisms are regulated by cytosolic calcium levels (*49*, *50*). By performing functional attachment/detachment assays and comparing surface expression of adhesion molecules, we found that *Cacnb3*-deficient BMDCs have an impaired ATP-dependent down-regulation of CD11b and CD44. Whereas CD11b is mainly regulated by calcium-controlled receptor recycling (*51*), it has been shown in cancer cells that the extracellular domain of CD44 is also cleaved in a calcium-dependent manner (*52*). However, the exact mode of action of CD11b and CD44 regulation in BMDCs remains currently unclear. Furthermore, we only analyzed well-described adhesion molecules in migDCs and therefore cannot exclude that other less-studied adhesion molecules also contribute to ATP-dependent detachment.

Our study has some limitations that are worth noting. We did not identify the surface receptor that induces the ATP-dependent calcium release. However, we could show that the signaling pathway involves PLC and IP_3_R and is therefore suggestive for being P2RY dependent. Furthermore, we did not explore other extracellular adenine nucleotides, which could also serve as DAMPs and induce a migratory phenotype in DCs. We did not specifically look at CACNB3-dependent differences in ADO signaling, which acts as an immunomodulatory counterpart of ATP.

In summary, we identified CACNB3 as a master regulator of ATP-dependent migDC migration. We provide insights into ATP-induced migDC signaling and show that CACNB3 regulates the IP_3_R-dependent calcium release from the ER. This signaling determines surface expression of adhesion molecules and therefore regulates detachment and initiation of migration. Further research is warranted to investigate the role of CACNB3 in migDCs in the context of disease-associated tissue damage.

## METHODS

### Mice

All mice [C57BL/6J, WT (The Jackson Laboratory); C57BL/6N, *Cacnb3^−/−^* (*37*); C57BL/6N, *LifeAct-GFP* and *LifeAct-GFP*;*Cacnb3^−/−^*] were kept under specific pathogen–free conditions in the central animal facility of the University Medical Center Hamburg-Eppendorf (UKE). We used adult mice (6 to 20 weeks old) from both sexes; mice were sex- and age-matched in all experiments. We did not observe sex-specific differences in any of the experiments; therefore, the sexes were reported together.

### migDC marker identification

GSE37448 was downloaded from Gene Expression Omnibus. Differential expression analysis was performed using the standard limma pipeline (*53*). DCs with high major histocompatibility complex II (MHCII) and CD11c expression from skin and organ dLNs were summarized as migDCs. The transcriptome of migDCs was contrasted to any other cell population. migDC marker genes were defined as genes that were differentially up-regulated in migDCs in each comparison. Heatmaps were generated using pheatmap package and *t*-distributed stochastic neighbor embedding (tSNE) plots by Rtsne package.

### Generation of BMDCs

We obtained the bone marrow from 6- to 12-week-old mice as described previously (*7*). Cells were homogenized through 40-μm cell strainers (Greiner) and incubated in red blood cell lysis buffer (0.15 M NH_4_Cl, 10 mM KHCO_3_, and 0.1 mM Na_2_EDTA in double-distilled H_2_O at pH 7.4) for 5 min. We cultured the remaining cells in 100-ml cell culture flasks (Sarstedt) in mouse complete medium containing granulocyte-macrophage colony-stimulating factor (GM-CSF; 20 ng ml^−1^) (PeproTech). We changed the medium every second day by carefully replacing the supernatant with fresh medium containing GM-CSF (20 ng ml^−1^). We harvested semiadherent BMDCs on days 6 to 8, unless stated otherwise. For microchannel analysis only, we prepared BMDCs as described previously (*7*, *22*, *54*).

### migDC sorting

Inguinal LNs were harvested from euthanized mice, single-cell suspension was obtained, and flow cytometric cell sorting into complete medium with 20% fetal bovine serum was performed. Three animals per bioreplicate were pooled. Subsequently, cells were washed twice with ice-cold phosphate-buffered saline (PBS), and cell pellets were snap-frozen on dry ice. RNA was purified using the RNeasy Mini Kit (QIAGEN). Representative gating strategies are shown in fig. S8.

### Primary mouse neuronal cultures

For primary cortical cultures, we euthanized pregnant C57BL/6J. We reserved tissue of each embryo for genotyping and isolated the cortex, dissociated, and plated cells at a density of 1 × 10^5^ cells per 1 cm^2^ on poly-d-lysine–coated wells (5 μM; Sigma-Aldrich, catalog no. A-003-M). Cells were maintained in neurobasal plus medium (supplemented with B27 plus, penicillin, streptomycin, and l-glutamine; Gibco, catalog no. A3582901) at 37°C, 5% CO_2_, and a relative humidity of 98%. If no cytarabine was applied, then cells were maintained in neurobasal medium (supplemented with B27, penicillin, streptomycin, and l-glutamine; Gibco). Cultures were harvested 21 days in vitro for RNA sequencing.

### mRNA sequencing

RNA sequencing libraries were prepared using the TruSeq stranded mRNA Library Prep Kit (Illumina) according to the manufacturer’s manual (document 1000000040498 v00) with a minimum total RNA input of 150 ng per sample. Libraries were pooled and sequenced on a NovaSeq 6000 sequencer (Illumina) generating 50–base pair paired-end reads. The reads were aligned to the Ensembl mouse reference genome (GRCm39) using STAR v2.4 (*55*) with default parameters. The overlap with annotated gene loci was counted with featureCounts v1.5.1 (*56*). Differential expression analysis was performed with DESeq2 (v3.12) (*57*) calling genes with a minimal twofold change and false discovery rate–adjusted *P* < 0.05 differentially expressed. Gene lists were annotated using biomaRt (v4.0) (*58*). Gene set enrichment analysis was performed using the clusterprofiler package (*59*).

### In vivo DC migration

We anesthetized mice for 5 min and painted their ears with 30 μl of 1% FITC (Sigma-Aldrich) in a carrier solution of acetone/dibutyl phthalate (1:1; Sigma-Aldrich and J. T. Barker). After 20 hours, we collected ipsilateral draining cervical LNs and contralateral nondraining cervical LNs and obtained single-cell suspensions. They were stained for CD11c and analyzed by flow cytometry after LIVE/DEAD staining. For ear tape stripping, we stripped an ear of WT or *Cacnb3^−/−^* mice 10 times with ordinary adhesive tape. After 20 hours, we collected ipsilateral draining and contralateral nondraining cervical LNs and quantified the number of migrated migDCs by flow cytometry as described above.

### Competitive DC migration in vivo

We concentrated BMDCs of respective genotypes at 2 × 10^7^ cells ml^−1^ in PBS and labeled them with either 2.5 μM carboxyfluorescein succinimidyl ester (Invitrogen) or 2.5 μM eFluor 670 (eBioscience) for 10 min at 37°C before stopping the reaction with ice-cold mouse complete medium for 5 min on ice. We stimulated BMDCs with LPS (100 ng ml^−1^) or 500 μM ATP for 30 min at 37°C, washed them twice, mixed them in equal numbers, and adjusted the cells to a concentration of 6 × 10^7^ cells ml^−1^. To rule out dye-specific effects, in each experiment, cells from both genotypes were labeled vice versa, and a third mix with cells labeled in both colors was added as control. We injected 20 μl of the mix in the footpad of C57BL/6 mice and, after 18 hours, collected the draining popliteal and nondraining inguinal LNs and analyzed migrated cells by flow cytometry. For each genotype, we calculated the homing index as previously described (*7*).

### Migration speed measurement in microchannels

Polydimethylsiloxane (PDMS; GE Silicones) was used to prepare 4-μm by 5-μm microchannels from a custom-made mold. The PDMS chamber and a 35-mm glass-bottom dish (World Precision Instruments, FD35-100) were plasma-activated before being bound to each other. The binding was left to strengthen in a 70°C oven for 1 hour. The microchannels were then plasma-cleaned, coated with fibronectin (10 μg ml^−1^; Sigma-Aldrich) at room temperature for 1 hour, and then washed three times with PBS before cell loading. We prepared microchannels as described previously (*22*, *54*). Briefly, PDMS (GE Silicones) was used to prepare 4-μm by 5-μm microchannels. We coated their surface with bovine plasma fibronectin (10 μg ml^−1^; Sigma-Aldrich) for 1 hour and then washed the surface three times with PBS before seeding of 1 × 10^5^ BMDCs in complete medium containing GM-CSF (50 ng ml^−1^). We imaged migrating BMDCs for 16 hours with a DMi8 inverted microscope (Leica) at 37°C in a 5% CO_2_ atmosphere and with a 10× dry objective lens (numerical aperture, 0.40 phase). A frequency of acquisition of one image per 2 min of transmission phase was used. We generated kymographs of the migrating cells by subtracting the mean projection of the whole movie to each frame, generating clear objects in dark background that were analyzed using a custom program, as we described previously (*22*, *54*).

### Actin distribution during migration

We performed BMDCs migration in microchannels as described above. Cells were obtained from LifeAct-GFP mice, allowing us to visualize their actin cytoskeleton in live migratory cells. For better F-actin visualization, we used 8-μm by 5-μm microchannels. Cells were imaged for 5 hours with a DMi8 inverted microscope (Leica) at 37°C in a 5% CO_2_ atmosphere with a 20× dry objective lens (numerical aperture, 0.75 phase). Movies of individual cells were cropped, aligned, and superposed to generate a mean projection representing the overall distribution of actin in cells migrating into the microchannels. For the statistical analysis of relative F-actin distribution in migrating cells, we calculated the intensity of phalloidin staining in the front (first thirds of the cell) and at the rear (the rest of the signal) on an individual cell level using Fiji software [National Institutes of Health (NIH)].

### DC adhesion

We coated 48-well flat-bottom, nontissue culture-treated plates with 100 μl of PBS containing 1% bovine serum albumin (PAA Laboratories) and bovine fibronectin (50 μg ml^−1^; Sigma-Aldrich) per well overnight at 37°C. We harvested BMDCs from *LifeAct-GFP*;WT and *LifeAct-GFP*;*Cacnb3^−/−^* mice and rested them in a serum-free medium containing GM-CSF (20 ng ml^−1^) for 2 hours at 37°C. Meanwhile, precoated plates were blocked with PBS containing 1% bovine serum albumin for 1 hour at 37°C. BMDCs were pulsed with LPS (100 ng ml^−1^) or 500 μM ATP, and subsequently, we plated 200 μl from 1 × 10^6^ cells ml^−1^ in complete mouse medium containing GM-CSF (20 ng ml^−1^) and incubated them for 2 hours at 37°C. Nonadherent cells were removed by washing them three times with serum-free medium. Images of living cells were acquired with a microscopy system within our incubator (IncuCyte). Image analysis was performed using Fiji software (NIH).

### Boyden chamber experiments

We used 3-μm Boyden chambers (Millipore, ECM505) in 24-well plates. BMDCs were harvested at day 6 or 8 as described above. They were pulsed in a density of 1 × 10^6^ cells ml^−1^ with 500 μM ATP, 100 nM LPS, 50 μM 2-APB, or 1 μM thapsigargin for 30 min. Subsequently, they were washed twice, and 100 μl of 1 × 10^6^ cells ml^−1^ were seeded in the Boyden chamber. CCL-19 (100 ng ml^−1^) and CCL-21 (100 ng ml^−1^) were used as chemoattractant. After 16 hours, cells in the 24-well plate were harvested, spun down at 300*g* for 10 min at 4°C, resuspended in 10 μl of ice-cold PBS, counterstained with trypan blue, and counted using a Neubauer counting chamber.

### Real-time PCR

We reverse-transcribed RNA to complementary DNA with the RevertAid H Minus First Strand cDNA Synthesis Kit (Thermo Fisher Scientific) according to the manufacturer’s instructions. We analyzed gene expression by real-time PCR performed in the ABI Prism 7900 HT Fast Real-Time PCR System (Applied Biosystems) using TaqMan Gene Expression Assays (Thermo Fisher Scientific) for *Cacnb1* (Mm01306805_m1), *Cacnb2* (Mm00659092_m1), and *Cacnb3* (Mm00432244_m1). We calculated gene expression as 2^–Δ*C*t^ relative to TATA-box binding protein (Tbp) (mouse) or TBP (human) as the endogenous control.

### Calcium imaging

We seeded BMDCs on either ibidi 60 μ-Dish Quad (catalog no. 80411) or High (catalog no. 81158) with a glass bottom. We used Fluo-4 AM (Thermo Fisher Scientific; catalog no. 14201) to measure calcium levels. For that, we incubated BMDCs in medium with 5 μM Fluo-4 AM at 37°C for 30 min at 5% CO_2_. Then, cells were rinsed three times and left to equilibrate in imaging buffer [10 mM glucose, 140 mM NaCl_2_, 1 mM MgCl_2_, 5 mM KCl, and 20 mM Hepes (pH 7.4)] for at least 30 min before imaging. If indicated, then BMDCs were preincubated with 50 μM 2-APB, 10 μM U73122, and 50 μM dantrolene. We acquired images with an LSM 700 laser scanning confocal microscope (Zeiss) every 0.48 s with a ×20 magnification EC Plan-Neofluar 20/0.5 (Zeiss, 420350-9900) objective in an imaging chamber maintaining 37°C and 5% CO_2_. At the end of recording, we applied 10 μM ionomycin together with 10 mM CaCl_2_ to induce maximum standardized cellular calcium response that was used for normalization. Specific assay details and concentrations can be found in the respective figure legend. For data analysis, we measured mean fluorescence values of every cell using Fiji software (NIH) and normalized it to the maximal calcium response after ionomycin challenge (indicated as *F*/*F*_Max_). For each cell, we calculated maximal, minimal, mean, and AUC of the calcium response using a custom R script. For comparisons of maximal calcium responses after ATP stimulation, only cells that showed a sufficient increase >2 SDs of the baseline mean fluorescence intensity (MFI) were considered to exclude a bias by nonreacting cells. For calcium imaging with the ratiometric probe Fura-2, we loaded BMDCs in medium with 5 μM Fura-2 AM (Invitrogen, Oregon, USA) at 37°C for 45 min at 5% CO_2_. After loading, cells were washed two times with imaging buffer [140 mM NaCl, 4 mM KCl, 2 mM MgCl_2_, 10 mM Hepes, and 10 mM glucose (pH 7.4), adjusted with NaOH]. Experiments were performed on an inverted microscope Axiovert S100 equipped with Fluar 20×/0.75 objective (Zeiss, Oberkochen, Germany), a monochromator (polychrome V, Till-Photonics, Martinsried, Germany), and a cooled charge-coupled device camera (Andor Technology). Fura-2 signals were recorded after excitation at 340 and 380 nm with a charge-coupled device camera, and the emitted fluorescence was detected at >440 nm (Fura filter CHROMA, Olching, Germany). Data are shown as mean ratio F340 nm/F380 nm versus time.

### Measurement of IP_1_

The IP_1_ concentration, a stable metabolite of IP_3_, was measured using the IP-One AlphaLISA Detection Kit (PerkinElmer, AL3145) following the manufacturer’s protocol and as described previously (*44*). Briefly, WT and *Cacnb3^−/−^* BMDCs isolated from three mice of each genotype were seeded onto white opaque 384-well microplate at a density of 20,000 cells per well, cultured for 48 hours, and stimulated with ATP (500 μmol liter^−1^) in the presence or absence of U73122 (10 μmol liter^−1^). After fitting a standard curve using a nonlinear regression, the IP_1_ concentration in nanomoles per liter was interpolated.

### Statistical analysis

The statistical analyses applied during the bioinformatics analysis are detailed in the respective sections of the article. Flow cytometric data were analyzed by using FlowJo (LLC). Images were analyzed using Fiji software (NIH). Experimental data were analyzed within the R environment on a Mac OS X. Unless stated otherwise, the data are presented as means ± SEM, and differences between two experimental groups were determined using unpaired, two-tailed Student’s *t* tests and were false discovery rate–corrected for multiple comparisons. The exact number of experiments is provided in the figure legends. Significant results are indicated by *P < 0.05, **P < 0.01, ***P < 0.001, and ****P < 0.0001.

### Study approval

All animal care and experimental procedures were performed according to institutional guidelines and conformed to the requirements of the German Animal Welfare Act. Ethical approvals were obtained from the State Authority of Hamburg, Germany (approval no. 15/81, 122/17).
