# Supplementary material for: Calcium channel β3 subunit regulates ATP-dependent migration of dendritic cells
Source: Sci Adv. 2023 Sep 20;9(38):eadh1653. doi: 10.1126/sciadv.adh1653 (PMC10511199; doi:10.1126/sciadv.adh1653)
Supplement: Supplementary file 1 — Figs. S1 to S8 Table S1 [file sciadv.adh1653_sm.pdf]

Science Advances
AAAS

Supplementary Materials for

Calcium channel β3 subunit regulates ATP-dependent migration of dendritic cells

Marcel S. Woo *et al.*

Corresponding author: Manuel A. Friese, manuel.friese@zmnh.uni-hamburg.de

Sci. Adv. **9**, eadh1653 (2023)
DOI: 10.1126/sciadv.adh1653

This PDF file includes:

Figs. S1 to S8
Table S1

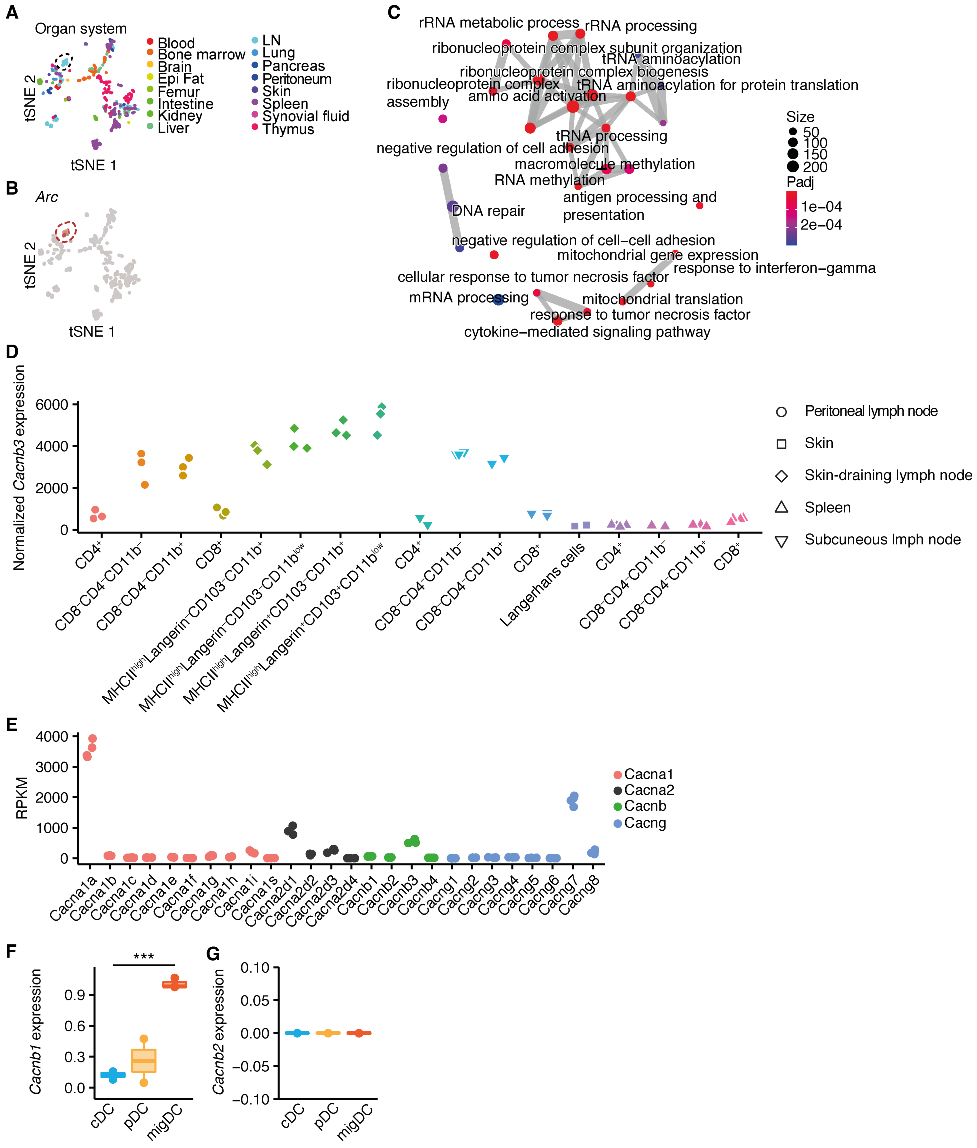

Fig. S1. migDCs are defined by CACNB3 expression. (**A**) tSNE representation of all included 653 samples from the ImmGen database (GSE15907) (25–27). Color indicates the annotated organ system from which the samples were originally retrieved. (**B**) tSNE representation of all included 653 samples from the ImmGen database (GSE15907). migDCs are highlighted, color shows *Arc* expression. (**C**) GO term biological process enrichment analysis of migDC-defining genes. Color shows adjusted *P*–values, size shows number of genes. (**D**) Normalized expression of *Cacnb3* in indicated DC subsets in lymph nodes, spleen and skin retrieved from the ImmGen database (GSE15907), color indicates different subsets, shape represents organs where cells were sorted from. (**E**) Reads per kilobase of transcript per million mapped reads (RPKM) of voltage-gated calcium channel subunits from primary neuronal cultures in RNA-sequencing ($n = 3$). (**F and G**) Relative expression of *Cacnb1* (F) and *Cacnb2* (G) in sorted cDCs, pDCs, and migDCs. If not stated otherwise unpaired *t*-test was used for statistical comparison, $***P < 0.001$.

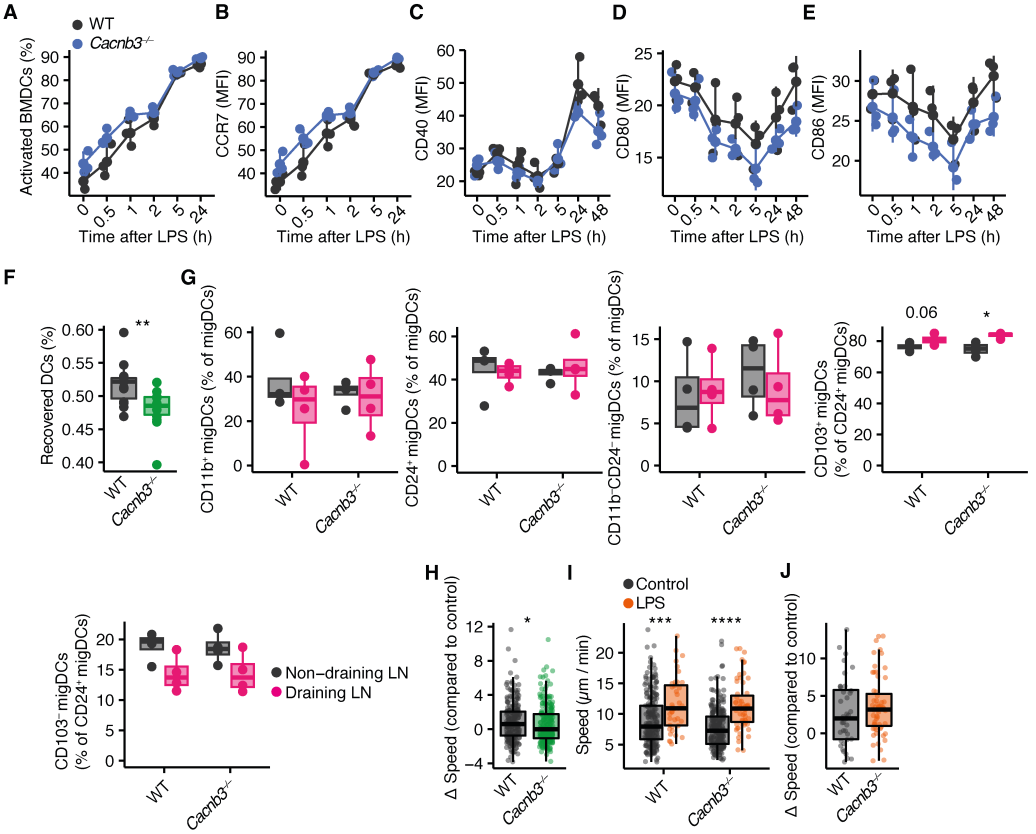

Fig. S2. LPS-induced activation and migration of BMDCs is independent of CACNB3. (**A to E**) Percentage of MHCII high BMDCs (A), CCR7 MFI (B), CD40 MFI (C), CD80 MFI (D) and CD86 MFI (E) of BMDCs from WT and *Cacnb3*$^{-/-}$ animals (n = 3) at different time points after stimulation with 100 ng mL^{-1} LPS. (**F**) Relative frequency of recovered migDCs in ipsilateral cervical lymph nodes after ear tape stripping experiment #2 (n = 9). (**G**) Percentage of different migDC subsets in draining and non-draining cervical lymph nodes after ear tape-stripping in WT and *Cacnb3*$^{-/-}$ animals (n = 4). (**H**) Speed difference of ATP-stimulated BMDCs from WT and *Cacnb3*$^{-/-}$ animals in comparison to vehicle-stimulated controls. (**I**) Quantification of mean speed (µm min^{-1}) of WT and *Cacnb3*$^{-/-}$ BMDCs that were imaged in microchannels after pulsing with vehicle or 100 ng mL^{-1} LPS. (**J**) Speed difference of LPS-stimulated BMDCs from WT and *Cacnb3*$^{-/-}$ animals in comparison to vehicle stimulated controls. If not stated otherwise unpaired *t*-test was used for statistical comparison, $^{*}P < 0.05$, $^{**}P < 0.01$, $^{***}P < 0.001$, $^{****}P < 0.0001$.

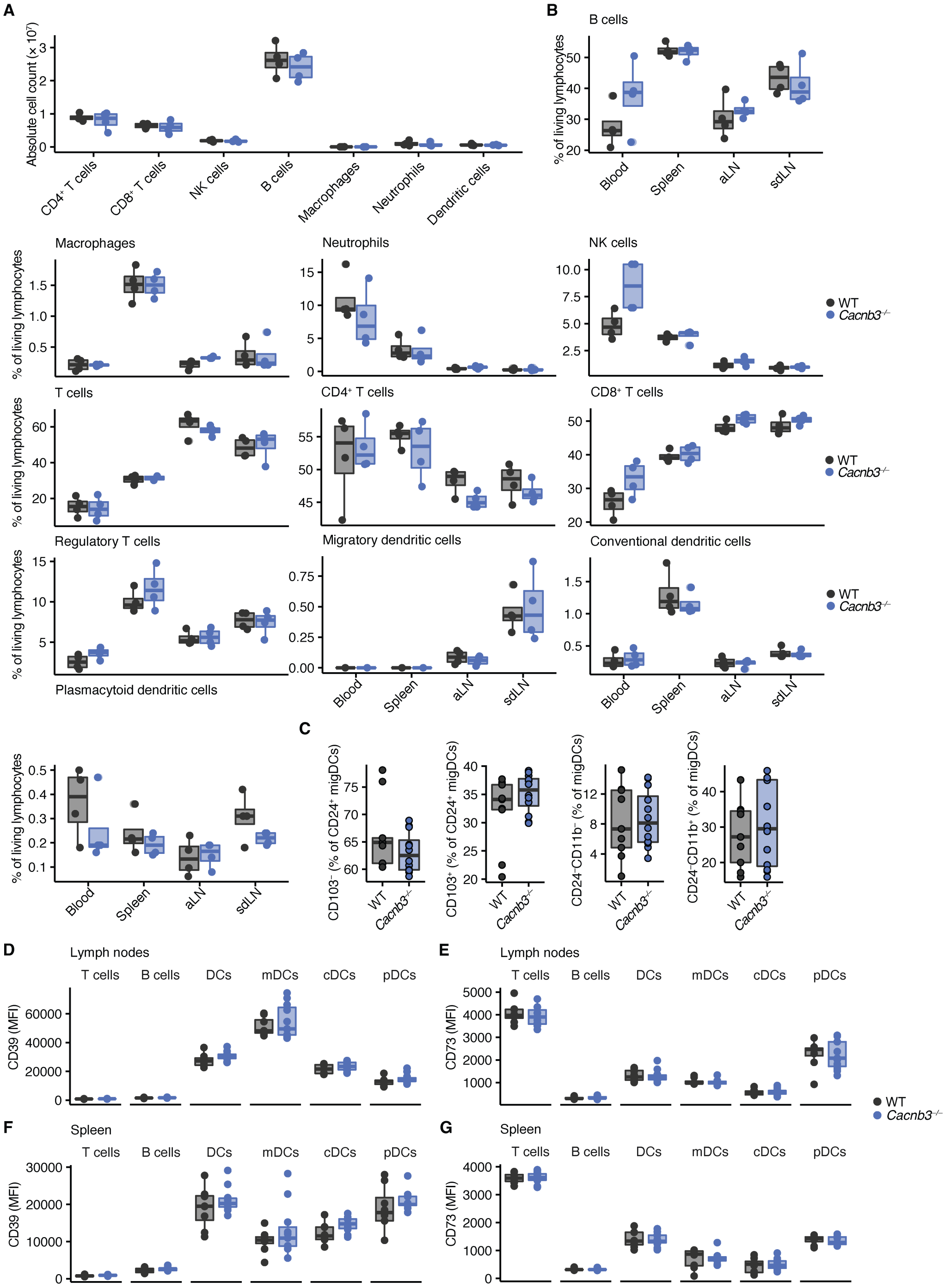

Fig. S3. Immune cell composition is not changed in *Cacnb3*-deficient animals in steady-state. (**A**) Absolute numbers of immune cell subsets in the spleens of healthy WT and *Cacnb3*$^{-/-}$ mice (n = 4). (**B**) Relative frequencies of immune cell subsets in blood, spleen, aortic lymph nodes (aLN), skin-draining lymph nodes (sdLN) of WT and *Cacnb3*$^{-/-}$ mice (n = 4). (**C**) Relative percentage of shown migDC subsets isolated from sdLNs of healthy WT (n = 9) and *Cacnb3*$^{-/-}$ mice (n = 12). (**D** and **E**) CD39 MFI (D) and CD73 MFI (E) in sdLNs nodes of WT (n = 6) and *Cacnb3*$^{-/-}$ (n = 8) mice of respective immune cell subsets. (**F** and **G**) CD39 MFI (F) and CD73 MFI (G) in spleens of WT (n = 6) and *Cacnb3*$^{-/-}$ (n = 8) of shown immune cell subsets. If not stated otherwise unpaired *t*-test was used for statistical comparison.

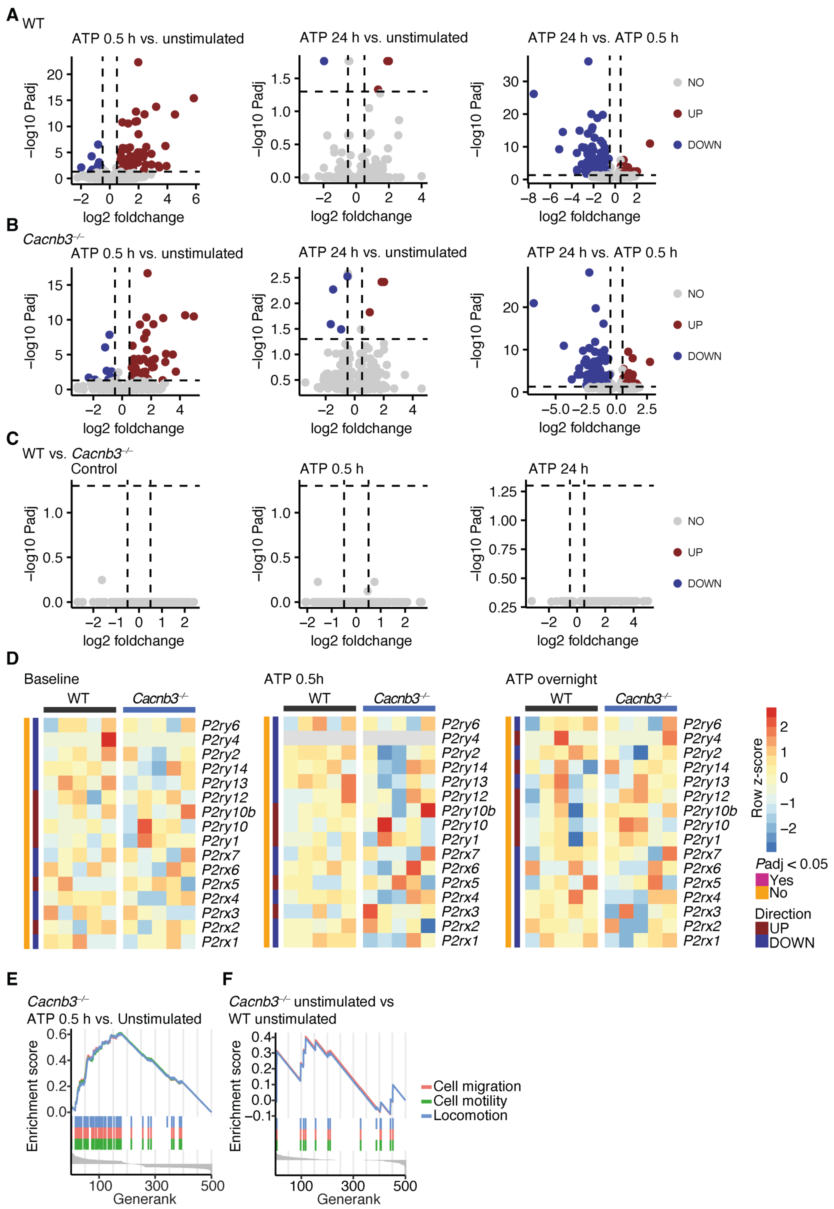

Fig. S4. No differential gene expression between WT and *Cacnb3*-deficient BMDCs. (**A** to **C**) Volcano plots depicting RNA sequencing results of WT (A) and *Cacnb3*$^{-/-}$ (B) BMDCs after 30 min pulse with 500 µM ATP and 24 h after ATP pulse and comparisons between genotypes (C). The comparisons are detailed in the figure. Differentially up- or downregulated genes with *P*adj < 0.05 and log2 foldchange > 0.5 or < –0.5 are colored in the plots. (**D**) Expression of all identified P2XR and P2YR in our RNA-sequencing datasets. Comparisons between WT and *Cacnb3*$^{-/-}$ BMDCs at baseline (left), directly after (middle) or after overnight incubation (right) following 30 min pulse with 500 µM ATP are shown. Color shows row z-score. No significant differences were found. (**E**) GO term enrichment analyses of GO terms “Cell migration” (NES = 1.58, *P*adj = 0.002), “Cell motility” (NES = 1.57, *P*adj = 0.002) and “Locomotion” (NES = 1.58, *P*adj = 0.001) in *Cacnb3*$^{-/-}$ BMDCs after 30 min ATP pulse in our RNA-sequencing experiment. All GO terms *P*-value < 0.01. (**F**) GO term enrichment analyses of GO terms “Cell migration” (NES = –1.4, *P*adj = 0.42), “Cell motility” (NES = –1.33, *P*adj = 0.46) and “Locomotion” (NES = –1.23, *P*adj = 0.52) in differentially expressed genes between WT and *Cacnb3*$^{-/-}$ BMDCs in our RNA-sequencing experiment. All GO terms *P*-value > 0.05.

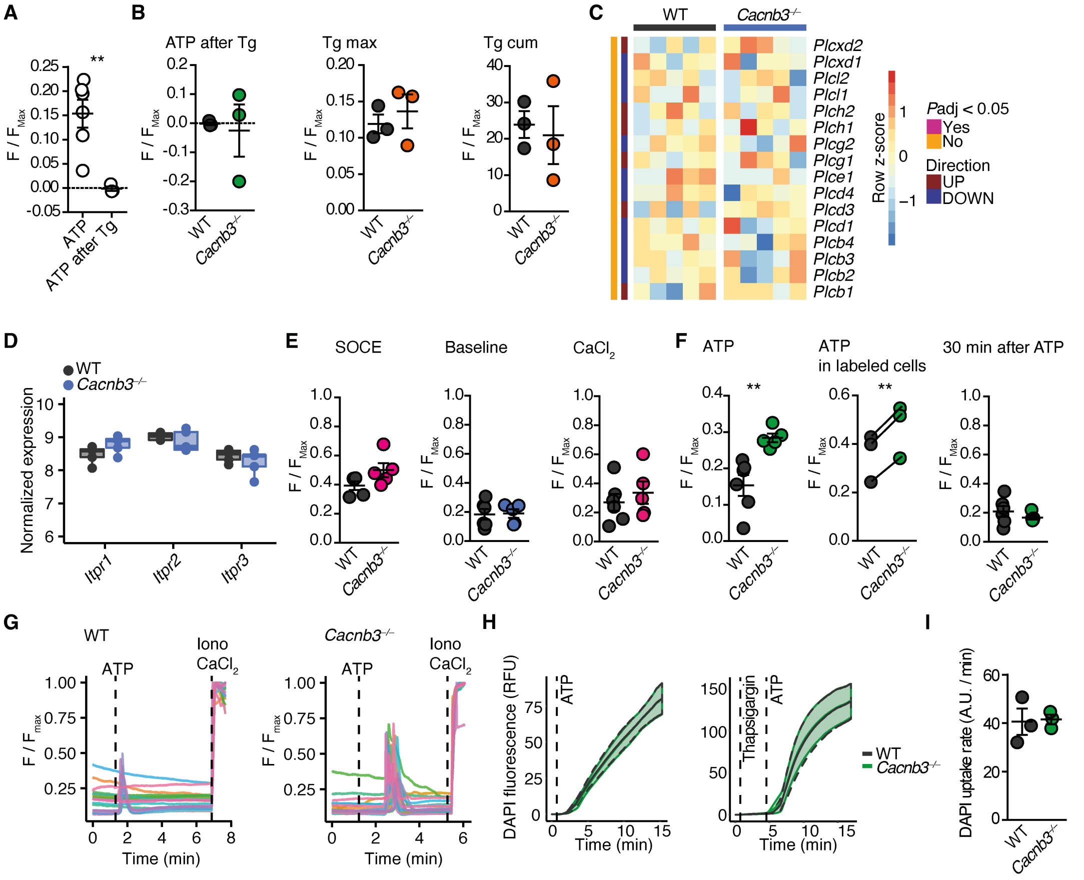

Fig. S5. Cacnb3 deficiency does not change ATP-induced DAPI uptake. (**A**) Maximal calcium response after 500 µM ATP stimulation alone or 500 µM ATP stimulation after 1 µM thapsigargin (Tg) application. (**B**) Comparison of maximal calcium response of WT and *Cacnb3*$^{-/-}$ BMDCs after 500 µM ATP stimulation after application of 1 µM thapsigargin (Tg; left). Maximal (middle) and cumulative (right) calcium response after stimulation with 1 µM thapsigargin (Tg) of WT and *Cacnb3*$^{-/-}$ BMDCs. (**C**) Gene expression of PLC subunits in steady-state WT and *Cacnb3*$^{-/-}$ BMDCs from our RNA-sequencing data. None of the depicted genes were differentially expressed. Color shows row z-score. (**D**) Normalized expression from our RNA-sequencing experiment of *Itpr1*, *Itpr2* and *Itpr3* in WT and *Cacnb3*$^{-/-}$ BMDCs (n = 5). (**E**) Maximal calcium response after store-operated calcium entry (SOCE) activation of Fluo-4 loaded WT and *Cacnb3*$^{-/-}$ BMDCs by subsequent depletion of calcium with 2 mM EGTA and 1 µM thapsigargin for 10 minutes and subsequent application of 2 mM calcium chloride (left), at baseline (middle) or application of calcium 2 mM $CaCl_2$ (right). (**F**) Maximal calcium responses of Fluo-4 loaded WT and *Cacnb3*$^{-/-}$ BMDCs that were stimulated with 500 µM ATP (left; n = 5), and of *Cacnb3*$^{-/-}$ BMDCs that were fluorescently labeled and mixed in a 1:1 ratio and were stimulated with 500 µM ATP (middle; n = 3), and calcium signal 30 minutes after stimulation with 500 µM ATP (right; n = 5). Paired *t*-test was used for comparison of calcium levels in labeled cells. (**G**) Representative calcium traces of 20 randomly selected cells from WT (left) and *Cacnb3*$^{-/-}$ (right) BMDCs that were loaded with Fluo-4 and were exposed to 500 µM ATP, F/F_{max} are shown. (**H**) Pooled traces of DAPI fluorescence of living WT and *Cacnb3*$^{-/-}$ BMDCs after stimulation with 500 µM ATP alone or after application of 1 µM thapsigargin. (**I**) Quantification of area under the curve per minute of DAPI uptake after ATP stimulation of living WT and *Cacnb3*$^{-/-}$ BMDCs (n = 3). If not stated otherwise unpaired *t*-test was used for statistical comparison, **$P < 0.01$.

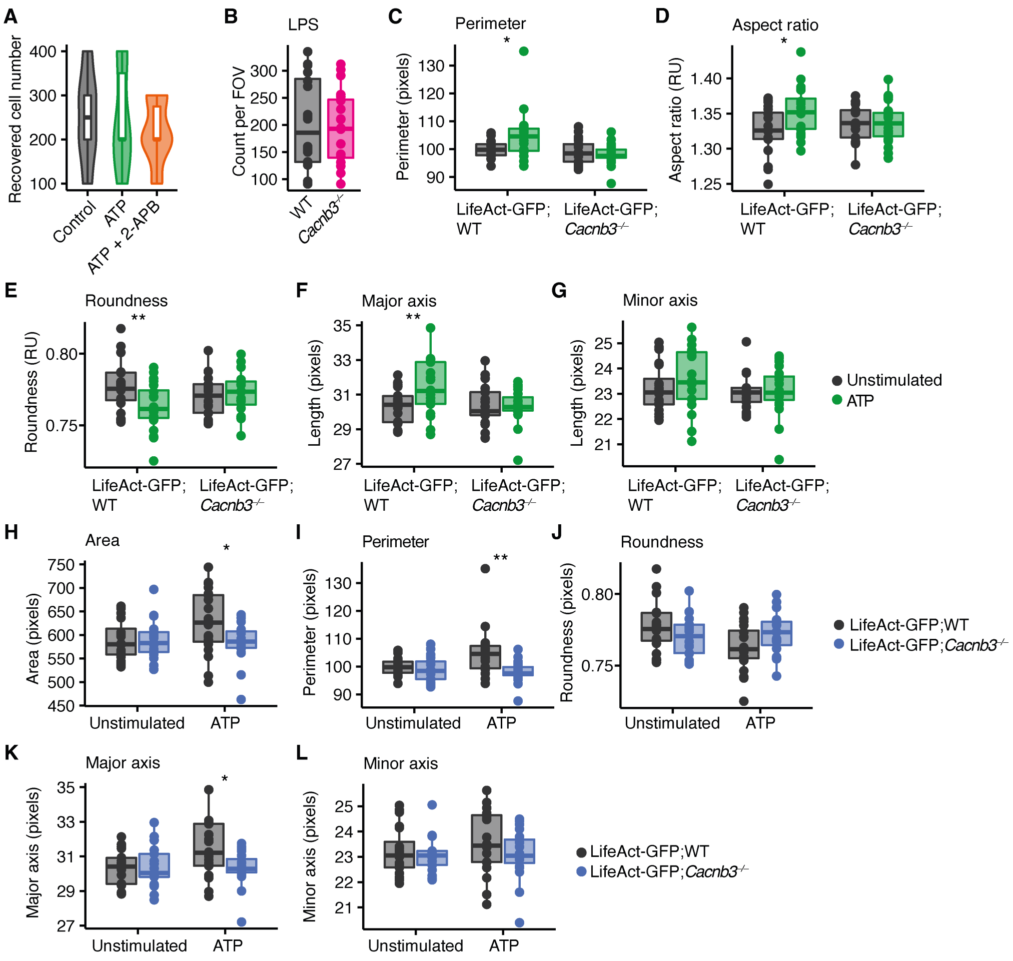

Fig. S6. Morphometric changes of WT and *Cacnb3*-deficient BMDCs after ATP stimulation. (**A**) Recovered cell number in Boyden chamber 2 h after stimulation with vehicle, 500 µM ATP 30 min pulse, 500 µM ATP pulse with 50 µM 2-APB and 1 µM thapsigargin ($n = 6$). (**B**) Count per FOV 6 h after 30 min pulse with 100 ng mL^{-1} LPS of WT and *Cacnb3*$^{-/-}$ BMDCs ($n = 20$). (**C** to **G**) Comparison of perimeter (C), aspect ratio (D), roundness (E), major axis (F), minor axis (G) after vehicle and 6 h after a 30 min pulse with 500 µM ATP of BMDCs from *LifeAct-GFP*;WT and *LifeAct-GFP*;*Cacnb3*$^{-/-}$ mice ($n = 20$). Comparisons between unstimulated and ATP stimulated BMDCs within respective genotypes are shown. (**H** to **L**) Comparison of perimeter (H), aspect ratio (I), roundness (J), major axis (K), minor axis (L) of BMDCs from *LifeAct-GFP*;WT and *LifeAct-GFP*;*Cacnb3*$^{-/-}$ mice after vehicle and 6 h after 30 min pulse stimulation with 500 µM ATP ($n = 20$). Comparisons between genotypes in vehicle- or ATP-stimulated BMDCs are shown. If not stated otherwise unpaired *t*-test was used for statistical comparison, $*P < 0.05$, $**P < 0.01$, $***P < 0.001$, $****P < 0.0001$.

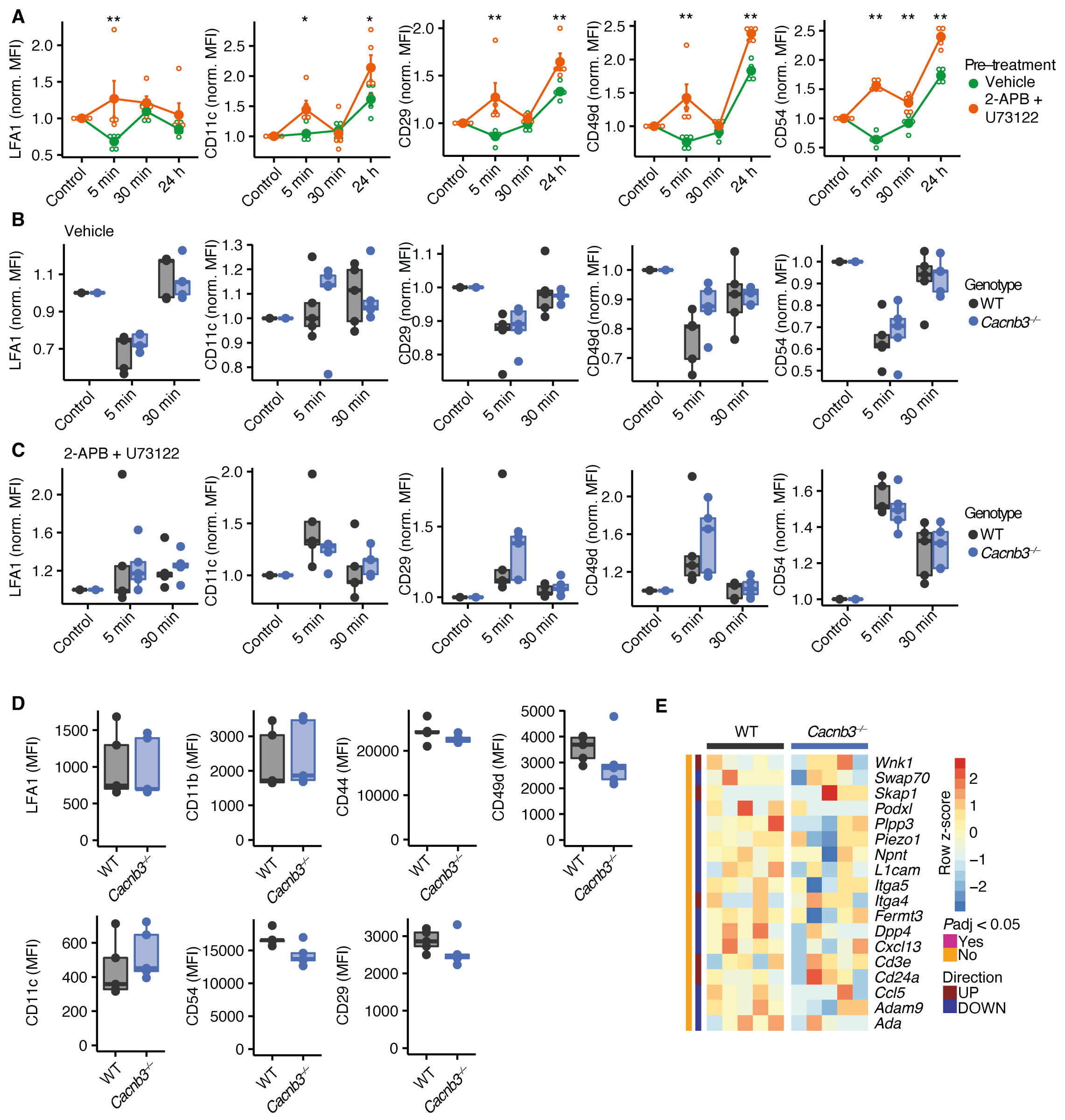

Fig. S7. Dynamic changes of adhesion molecule surface expression after ATP stimulation. (**A**) MFI of LFA1, CD11c, CD29, CD49d, CD54 after vehicle stimulation or 5 min, 30 min or 24 h after stimulation with 500 µM ATP or 500 µM ATP together with 50 µM 2-APB and 1 µM U73122 (*n* = 5). (**B**) Normalized MFI of LFA1, CD11c, CD29, CD49d, CD54 after vehicle stimulation or 5 min, 30 min or 24 h after stimulation with 500 µM ATP in WT and *Cacnb3*$^{-/-}$ BMDCs (*n* = 5). Data were normalized to controls of each genotype. (**C**) Normalized MFI of LFA1, CD11c, CD29, CD49d, CD54 after vehicle stimulation or 5 min, 30 min or 24 h after stimulation with 500 µM ATP with 50 µM 2-APB and 1 µM U73122 in WT and *Cacnb3*$^{-/-}$ BMDCs (*n* = 5). Data were normalized to controls of each genotype. (**D**) Raw MFI of LFA1, CD11b, CD44, CD49d, CD11c, CD54 and CD29 in WT and *Cacnb3*$^{-/-}$ BMDCs (*n* = 5) at steady-state. (**E**) Expression of depicted adhesion molecules in RNA-sequencing dataset of WT and *Cacnb3*$^{-/-}$ BMDCs at steady-state. Color shows row z-score. If not stated otherwise unpaired *t*-test was used for statistical comparison, *$P < 0.05$, **$P < 0.01$.

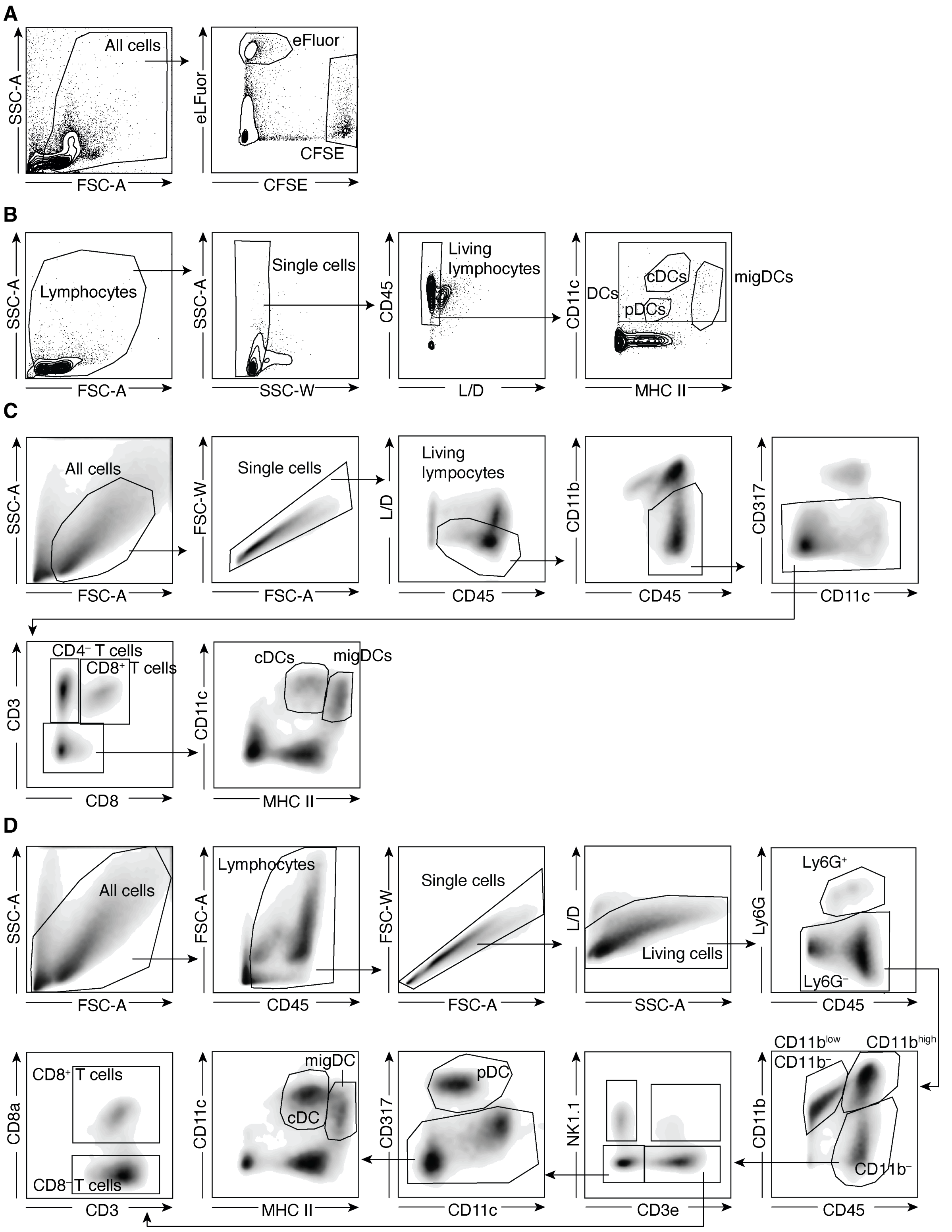

Fig. S8. Representative gating strategies. (**A**) Identification of labeled BMDCs after footpad injection. (**B**) Sorting strategy for different DC populations. (**C**) Identification of DCs by flow cytometry. (**D**) Peripheral immune cell phenotyping.

Table S1. List of antibodies used in this study.

Antigen	Host species	Company	Catalogue no.	Ig fraction	Fluorophore	Dilution FACS
**CACNB3**	Rabbit	generated in-house		IgG		
**CD3ε**	Armenian hamster	BioLegend	100305	IgG	FITC	1:100
**CD8a**	Rat	BioLegend	100750	IgG	BV785	1:200
**CD11b**	Rat	BD Biosciences	563553	IgG	BUV395	1:200
**CD11b**	Rat	BioLegend	101228	IgG	PerCP-Cy5.5	1:400
**CD11c**	Armenian hamster	BioLegend	117336	IgG	BV786	1:300
**CD11c**	Armenian hamster	BioLegend	117318	IgG	PE-Cy7	1:300
**CD19**	Rat	BioLegend	115541	IgG	BV650	1:400
**CD19**	Rat	BioLegend	115506	IgG	FITC	1:100
**CD29**	Armenian Hamster	BioLegend	102205	IgG	FITC	1:200
**CD39**	Rat	BioLegend	143808	IgG	AF647	1:300
**CD40**	Rat	BioLegend	124612	IgG	APC	1:100
**CD44**	Rat	BioLegend	103049	IgG	BV650	1:200
**CD45**	Rat	BioLegend	103127	IgG	AF700	1:200
**CD45**	Rat	BioLegend	103112	IgG	APC	1:200
**CD49d**	Rat	BioLegend	103608	IgG	PE	1:100
**CD54 (ICAM-1)**	Rat	BioLegend	116121	IgG	PE-Cy7	1:400
**CD73**	Rat	BioLegend	127206	IgG	PE	1:200
**CD80**	Armenian hamster	BioLegend	104705	IgG	FITC	1:100
**CD86**	Rat	BD Biosciences	750437	IgG	BUV496	1:200
**CD197**	Rat	BioLegend	120105	IgG	PE	1:100
**CD205**	Rat	BioLegend	138213	IgG	PE	1:200
**CD272 (BTLA)**	Rat	BioLegend	134809	IgG	PE-Cy7	1:100
**CD274 (PD-L1)**	Rat	BioLegend	124311	IgG	BV650	1:100
**CD279 (PD-1)**	Rat	BioLegend	135224	IgG	APC	1:100
**CD317 (PDCA1)**	Rat	BioLegend	127016	IgG	APC	1:100
**CD317 (PDCA1)**	Rat	BioLegend	127103	IgG	PE	1:100
**F4/80**	Rat	BioLegend	123110	IgG	BV421	1:100
**I-A/I-E**	Rat	BioLegend	107643	IgG	BV711	1:400
**I-A/I-E**	Rat	BioLegend	107620	IgG	PB	1:400
**LFA-1**	Rat	BioLegend	101114	IgG	AF647	1:100
**Ly6G**	Rat	BioLegend	127623	IgG	APC-Cy7	1:400
**Ly6G**	Rat	BioLegend	127605	IgG	FITC	1:200
**NK1.1**	Rat	Invitrogen	12-5941-82	IgG	PE	1:300
